# Effect of *Moringa oleifera, Bacillus amyloliquefaciens*, and Their Combination on Growth Performance, Digestive Enzymes, Immunity, and Microbiota in Nile Tilapia (*Oreochromis niloticus*)

**DOI:** 10.1155/2024/1755727

**Published:** 2024-03-04

**Authors:** Mingxuan Guan, Junfeng Guan, Haotian Zhang, Dikuang Peng, Xiaotong Wen, Xiaoyong Zhang, Qing Pan

**Affiliations:** College of Ocean, South China Agricultural University, Guangzhou 510642, Guangdong, China

## Abstract

To exploit aquatic animals' feed resources and functional ingredients and additives, this experiment investigated the effects (a 2 × 2 factorial trial) and mechanism of woody plants and probiotics on Nile tilapia. Each treatment group was randomly assigned triplicate nets of 40 fish (initial body weight of 35.04 ± 0.01 g) in 12 tanks for 56 days. Fish were fed four diets, including a CON–CON diet (with no supplementation) and CON–Ba (basal diet + 1 × 10^7^ CFU/g *B. amyloliquefaciens*), MO-CON (basal diet + 10% *Moringa oleifera*) and MO-Ba (basal diet + 1 × 10^7^ CFU/g *B. amyloliquefaciens* + 10% *Moringa oleifera*) until apparent satiation (2 meals/day). The results showed that the MO diet had a lower feed conversion ratio than the non-MO diets (*P* < 0.05). The weight gain in the Ba group was significantly higher than those in the groups without Ba treatment (*P* < 0.05). Both MO and Ba improved the morphological indices when applied individually (*P* < 0.05). Ba supplementation improved the activities of digestive enzymes (*P* < 0.05). MO significantly upregulated the gene expression of *TNF-α*, *IL-6*, *IL-10*, and *C3* in the spleen of Nile tilapia (*P* < 0.05), while Ba significantly upregulated the gene expression of *IL*-6 and *C3 (*P* < 0.05*). The microbiological analyses showed that the abundances of beneficial bacteria increased, and the relative abundances of pathogenic bacteria decreased in fish-fed MO. In conclusion, dietary MO and Ba can effectively improve the immunity of Nile tilapia, while Ba can enhance the activities of digestive enzymes. The synergistic effect of MO and Ba on the intestinal microbiota of Nile tilapia promotes growth, thus showing promising application prospects.

## 1. Introduction

Protein is the main substance that maintains the physiological activities of fish, while fish meal is widely used in aquafeeds. However, due to the gradual scarcity of fishery resources and the high price of fish meal, the development of the aquafeed industry has been restricted [1, 2]. Tilapia farming is the second largest aquaculture system in recent years after carp farming [3]. The long-term intensive cultivation of tilapia inevitably increases the probability of fish diseases, leading to environmental pollution and food safety issues [4]. The intensive farming mode makes tilapia susceptible to pathogenic bacterial infections, with the most harmful bacteria being *Streptococcus*, which results in high mortality [5]. To address the frequently occurring diseases caused by environmental deterioration, the use of antibiotics has also increased. The misuse of antibiotics in farming not only leads to antibiotic resistance in pathogenic bacteria but also poses a risk to human health. Finding potential natural alternatives to antibiotics has become an urgent issue that needs to be addressed [6]. Therefore, dietary supplementation with functional feed ingredients and probiotics seems to be a solution since it can enhance the health and growth performance of fish.

*Moringa oleifera* is richer in vitamin C than some fruits and plants [7]. According to related studies, 17 amino acids can be detected in the dried leaves of *M. oleifera* [8]. *M. oleifera* can thrive in tropical regions and has a wide range of adaptations to different ecological conditions, even drought and frost [9]. In addition, *M. oleifera* can be stored for a long time without losing its nutrients. The planting requirements of *M. oleifera* are not particularly strict, making it suitable for large-scale cultivation. *M. oleifera* is rich in flavonoids, polyphenols, terpenes, alkaloids, and other chemical substances. Two major components in MO flavonoids show a strong hypoglycemic effect [10]. Additionally, MO seed cake is often used as a natural biofloc in water treatment applications [11]. MO root extracts have also been proven to have the potential for inhibiting the growth of *Escherichia coli, Klebsiella pneumoniae*, and *Listeria monocytogenes*, which are pathogenic bacteria [12]. As an omnivorous fish, tilapia digestion is primarily concentrated in the intestine. Given that the intestines of tilapia are quite lengthy, they are well-suited to serving as a model fish species for the consumption of plant protein.

Probiotics have been studied as feed additives to improve fish utilization of plant protein-based feeds [13, 14]. Research has shown that probiotics can have a beneficial effect on the host by altering the structural composition of microbial colonies in the animal or farming environment [15]. Dietary probiotic (*Bacillus* and *Lactobacillus*) supplementation resulted in a significant enhancement of digestion via the improvement of the length of the intestine and had a substantial influence on growth performance [16]. *Bacillus amyloliquefaciens* is a Gram-positive bacterium with a rod shape that lives a parthenogenetic anaerobic life. In research on Nile tilapia, diets supplemented with *B. amyloliquefaciens* significantly increased the villus height in the intestine, thereby improving the efficiency of nutrients uptake by tilapia [17]. After feeding with *B. amyloliquefaciens*, the weight gain of Nile tilapia was significantly improved [18]. Notably, *B. amyloliquefaciens* mainly produces a series of metabolites during growth and reproduction. Therefore, further exploration of the composition and function of *B. amyloliquefaciens* is needed to assess its potential for use in aquaculture. To verify this hypothesis, this experiment was conducted to verify whether *B. amyloliquefaciens* has a positive effect on Nile tilapia.

The intestine can be regarded as a complex microbial ecosystem. In recent years, studies have been dedicated to studying the intestinal flora of various fish species, including zebrafish, *Clarias gariepinus*, and grass carp [19–21]. Studying the intestinal microbiota of fish will be beneficial in screening for bacteria that improve fish health. The microbial community in the fish intestine may be altered by consumed different feeds. For example, studies have shown that dietary woody forages can influence Nile tilapia gut microbiota [22]. Apart from different species, variations in microbial communities can also be found in different environments. Currently, many experiments are now investigating the impact of probiotics added to feed [23] or used as water additives [24] on microbial community structure, but the interaction between environmental microbiota and fish microbiota has yet to receive sufficient attention in the current literature. Here, to determine whether MO and Ba have an effect on microbiota, we compared the microbial structure of experimental pond water samples and Nile tilapia intestinal samples by using high-throughput sequencing.

To date, no experimental studies have been tested to investigate the effect of adding *M. oleifera* and *B. amyloliquefaciens* in combination with Nile tilapia feeds. Therefore, the aim of this study was to examine the individual and combined effects of MO and Ba on growth, feed utilization, digestive enzymes, immunity, and intestinal microbiota-associated environmental compartments in Nile tilapia.

## 2. Materials and Methods

### 2.1. Source of *M. oleifera* and Bacteria

Dry *M. oleifera* leaf powder (MO) was purchased from Alibaba (Zhejiang, China). The bacterial strain *B. amyloliquefaciens* (Ba) was provided by South China Agricultural University and was previously isolated from the intestine of Nile tilapia, which was identified by cluster analysis of the 16S rRNA sequence (SCAU-070, GenBank: MK281516.1) [25]. The intestinal samples were thoroughly with sterile water and then the bacterial fluid was spread onto prepared plates. The plates were cultured and selected according to the morphology to get the bacterial isolates. *B. amyloliquefaciens* was inoculated in a liquid medium (beef extract, 5.0 g; peptone, 10.0 g; yeast extract, 5.0 g; NaCl, 5 g; dextrose, 10.0 g; 1,000 ml of distilled water, adjusted pH at 7.0). Incubation was done at 28°C for 24 hr. After 24 hr, these cultures were centrifuged (Eppendorf AG 22,331 Hamburg) at 5,000 r/min for 10 min to remove the supernatant and washed twice with PBS (2.7 mM KCl; 137 mM NaCl; 1.8 mM KH_2_PO_4_; 10.1 mM NaH_2_PO_4_, pH 7.4). Then, the cultures were subjected to freeze–drying. Bacteria are preserved in freeze-dried form, and the bacteria strain activation was carried out before feed production. The bacterium was further cultured on De Man, Rogosa, and Sharpe agar to achieve a bacterial count of 1 × 10^7^ CFU/g.

### 2.2. Fish Culturing

Nile tilapia were obtained from the National Tilapia Breeding Farm of Guangdong Province, China. The experiments were conducted in Guangzhou, located at the Guangdong Academy Of Agricultural Sciences, China. The measured parameters were (30.86 ± 1.35°C) for the average temperature at 7:00 a.m., (33.14 ± 2.15°C) at 3:00 p.m., and (32.21 ± 1.98°C) at 6:00 p.m., and (8.0–8.8) for pH values. The fish were fed the basal diet (35% crude protein) during acclimatization. After 2 weeks of acclimatization, a total of 480 healthy Nile tilapia (initial mean weight 35.04 ± 0.05 g) were categorized into four groups in the earthen pond. Each tank was sized 1.5 m× 1.5 m × 1.5 m, and each tank was used to raise 40 fish. The experiment lasted 56 days. Fish were fed the diets to apparent satiation twice daily (8:00 a.m.; 5:00 p.m.), and the amount of feed fed was recorded daily. Uneaten feed was removed in time to calculate the feed conversion rate after drying. The feeding time and the amount of the diet were adjusted according to weather or emergency environmental situations. The fish mortality was observed and recorded daily. The water temperature of the pond is measured using a thermometer, and pH values are measured using a portable pH meter.

### 2.3. Diet Preparation

The elements and nutrient composition of the feed are depicted in Table 1. We determined that the appropriate amount of MO to be added to the diet was 10%, and the concentration of Ba to be added to the diet was 1 × 10^7^ CFU/g [22–25]. Soybean oil and wheat flour were adjusted accordingly. All raw materials were weighed in proportion and then added to oil and water for complete mixing after thorough crushing. The mixture was extruded into expanded pellet feed by a puffing machine (NH-10L, Guangzhou, China). *B. amyloliquefaciens* were mixed with the purple perilla seed oil and water to allow the bacteria to adhere to the feed. The feed was slowly and carefully added to the precultivated microbial solution while being stirred in a feed mixer (B20-Ⅱ, Hebei, China). The feed was placed in a hot-air oven (CT-C, Jiangsu, China) for drying and then sieved and sealed in Ziplock bags in the refrigerator. During extrusion, the temperature was kept below 100°C (the strain of SCAU-070 is capable of surviving below 100°C, as described in our previous study by a thermal resistance test). The feed was preserved at 4°C to keep the bacteria from losing viability.

### 2.4. Sample Collection

The Nile tilapia were fasted for 24 hr before sample collection, and the fish were anesthetized with Tricaine methanesulfonate (MS-222, 100 mg/mL). The final body weights of the experimental tilapia were recorded, and the number of individuals was counted to determine the survival rate. Then, nine fish from each replicate were dissected, among them, three fish were used for the proximate analysis of the whole-body composition, and another three fish were selected for measuring the morphological indices. Viscera, liver, stomach, and intestines were collected, and then morphological indices were calculated. The intestine (divided into foregut, midgut, and hindgut) and stomach samples were collected for digestive enzyme activity analysis. Additionally, spleen tissue samples and intestinal samples were collected and immediately frozen in liquid nitrogen. The other three fish were taken for sampling to obtain the intestine for intestinal microbiota analysis. We used a sterile water collector to collect the pond water samples. Each water sample size was 1 L [26]. Water samples were divided into three groups and filtered by using a GF/C filter with a pore size of 0.22 *μ*m. Finally, all samples were stored at −80°C for analysis. All samples were sent to Majorbio-Pharm Technology (Shanghai, China) for high-throughput sequencing.

To avoid interactions between bacteria-added and unsterilized diets, the tilapia fed with Ba diets (CON–Ba and MO–Ba) and the tilapia not fed with Ba diets (CON–CON and MO–CON) were placed in two separate cages that were located at a considerable distance from each other: (1) Intestinal samples were divided into four groups based on feed type. C_1–C_3 represented the tilapia fed the CON–CON diet; Cbs_1–Cbs_3 represented the tilapia fed the CON-Ba diet; M_1–M_3 represented the tilapia fed the MO-CON diet; Mbs_1–Mbs_3 represented the tilapia fed the MO-Ba diet. (2) To investigate the association of intestinal microbiota and water microbiota, the samples were divided into three groups as follows: S_1–S_3 served as the control group (water collected from the pond before starting experiment); SP_1–SP_3 served as the water in the area of CON-Ba and MO-Ba (water collected from the special area at 56 days); SW_1–SW_3 served as the water in the area of CON–CON and MO-CON (water collected from the special area at 56 days).

### 2.5. Chemical Analysis

The chemical proximate analysis of whole-body was performed at South China Agriculture University. The gross energy of diets was measured using the Calorimeter (Parr 6400, USA). The crude moisture content was measured using a warm air oven (GRX-91413, Shanghai) at 105°C until constant weight. The crude fat content was determined by the Soxhlet method with a Soxhlet extractor (ST 255 Soxtec™, FOSS, China). The crude ash content was determined by combusting dry samples in a muffle furnace at 550°C for 4 hr until reaching a constant weight. The crude protein was determined by the Kjeldahl method with an automatic Kjeldahl analyzer (KDN-19K, Shanghai) after acid digestion.

### 2.6. Digestive Enzyme Activity Analysis

Each intestinal and stomach sample was prepared for analysis of enzyme activity for the digestive enzyme activity (lipase (LPS), amylase (AMS), and protease (CAS), according to the manufacturer instruction) test kit (Nanjing Jiancheng Bioengineering Institute, Jiangsu, China).

### 2.7. Immunity Indices

The kits for the spleen levels of tumor necrosis factor *α* (*TNF-α*), interleukin 6 (*IL-6*), interleukin 10 (*IL-10*), and complement *C3* were purchased from Enzyme-Linked Biotechnology, Shanghai, China. An enzyme-linked immunosorbent assay (ELISA) kit, exclusively for tilapia, was used according to the manufacturer's instructions. The concentration of each sample was calculated by means of a standard curve and ELISA Calc V0.1 software.

### 2.8. Quantitative Real-Time q-PCR

Total RNA was extracted from spleen samples using a Tissue Total RNA isolation kit V2 (purchased from Vazyme, China). RNA concentrations were validated using a NanoDrop spectrophotometer (NDone, Gene Company Limited). After that, cDNA synthesis was performed by HiScriptRⅢ RT SuperMix for qPCR (procured from Vazyme). The synthesized cDNA samples were preserved at −20°C until use. Real-time PCR amplifications were performed using ChamQ Universal SYBR PCR Master Mix (procured from Vazyme) in 20 *μ*L reaction mixtures containing 2 *μ*L of cDNA, 0.8 *μ*L of the primer (10 *μ*M each) and SYBR 10 *μ*L. The Ct values of target genes were assessed by RT-PCR system (CFX96, Bio-Rad, USA). Primer sequences of the target mRNA of immune genes are described in Table 2. All primer sequences were designed using Premier 5.0. The PCR program consisted of denaturation at 95°C for 30 s, followed by 40 cycles at 95°C for 10 s, annealing at 60°C for 30 s, and a final extension step at 95°C for 15 s, 60°C for 1 min, 95°C for 15 s. The relative mRNA levels of the targeted genes in each sample were normalized to *β-actin* expression calculated by the comparative threshold cycle method. The relative expression levels were calculated by the 2^−*ΔΔ*CT^ method.

### 2.9. Bioinformatics Analysis

The quality of the extracted genomic DNA was detected using 2% agarose gel electrophoresis. PCR amplification was performed using TransGen AP 221-02, with 10 ng of DNA as the template and a 20 *μ*L reaction system, and using PCR primers for amplification of two highly variable regions of prokaryotic 16S rDNA, including V3 and V4. The V3–V4 regions were amplified by PCR (95°C for 3 min, followed by 30 cycles at 95°C for 30 s, 72°C for 30 s, 72°C for 45 s, and a final extension at 72°C for 10 min) using primers 338F (5′-ACTCCTACGGGAGGCAGCAG-3′) and 806R (5′-GGACTACHVHHHTWTCTAAT-3′). PCR reactions were performed in triplicate with 4 *μ*L of 5× FastPfu buffer, 2 *μ*L 2.5 mM dNTPs, 1.6 *μ*L of each primer (5 *μ*M), 0.4 *μ*L of FastPfu polymerase, 0.2 *μ*L of BSA and 10 ng of template DNA. PE reads were assigned to samples based on their special barcode and truncated by cutting off the barcode and primer sequence. Paired-end reads were merged using Flash 1.2.11. Using QIIME (system, 1.9.1.) to obtain high-quality tags. All raw tags were subjected to quality control and selection. The sequences were analyzed with Uparse software (system, 7.0.1090), and all sequences were assigned to the same OTUs (with the sequences over 97% similarity). During the clustering process, chimera were removed to obtain representative sequences for each OTU. RDP Classifier (system 2.13) was used to annotate the taxonomic information. Alpha diversity indices were calculated with the QIIME system. The difference in microbial composition between intestinal and water samples was assessed according to the Kruskal–Wallis *H* test. Hierarchical analysis was performed by the distance matrix of beta diversity. The calculation of beta diversity was performed by principal coordinates analysis (PCoA). Functional profiling was performed by PICRUSt1, and functional prediction was subsequently performed using the KEGG (Kyoto Encyclopedia of Genes and Genomes) pathway. Statistical analysis was carried out by the Wilcoxon rank-sum test (Wilcoxon).

### 2.10. Statistical Analyses

The data are expressed as the mean ± standard error of the mean (SEM). A two-way ANOVA was used to test the effect (whether significant or not) of *M. oleifera* and *B. amyloliquefaciens* alone or in combination on Nile tilapia by using SPSS Statistic version 21.0 software (SPSS Inc, Chicago, IL, USA). A probability value of *P* < 0.05 was considered statistically significant. Tukey's test was selected for comparisons of significance between four groups when there is a significant interaction effect between *M. oleifera* and *B. amyloliquefaciens*.

## 3. Results

### 3.1. Growth Performance

The survival rate of each group was above 88% and unaffected by the treatment (Table 3). Due to the differences in feed intake, supplementation with MO reduced the FBW, WGR, and SGR but increased the PER and decreased the FCR (*P* < 0.05; Table 3). Fish in group MO–CON had the lowest FCR (0.95). Ba supplementation significantly improved the FBW, WGR, and SGR (*P* < 0.05; Table 3). Compared with CON–CON and CON–Ba, MO–Ba resulted in a greater enhancement in growth performance.

### 3.2. Morphological Indices

As shown in Table 4, VSI, GSI, and CF were markedly improved with dietary MO and Ba (*P* < 0.05; Table 4). For the morphological indices (VSI and GSI), there was an interaction effect between MO and Ba (*P* < 0.05).

### 3.3. Whole-Body Composition

The whole-body composition is given in Table 5. The crude moisture of tilapia was unaffected by the treatment (*P* > 0.05). A significant increase in protein and ash content was observed with MO supplementation (*P* < 0.05), but the crude fat content was significantly reduced. Supplementation with Ba significantly increased the whole-body crude fat but significantly reduced the ash content of the whole-body (*P* < 0.05). There was an interaction effect between MO and Ba on whole-body crude moisture and ash content (*P* < 0.05).

### 3.4. Digestive Enzymes

Digestive enzymes are given in Table 6. Dietary supplementation with MO had no significant effect on the intestinal digestive enzyme activities of Nile tilapia. MO significantly reduced the activities of CAS in the stomach of Nile tilapia (*P* < 0.05). Ba can significantly increase the activities of AMS and LPS in the foregut and hindgut of fish, while also significantly increasing the activities of CAS of the stomach (*P* < 0.05).

### 3.5. Immunological Variables

Immunological variables in the spleen are given in Table 7. The contents of *TNF-α* and *C3* in the spleen were significantly increased with MO supplementation (*P* < 0.05).

### 3.6. mRNA Expression

**Figure 1:**
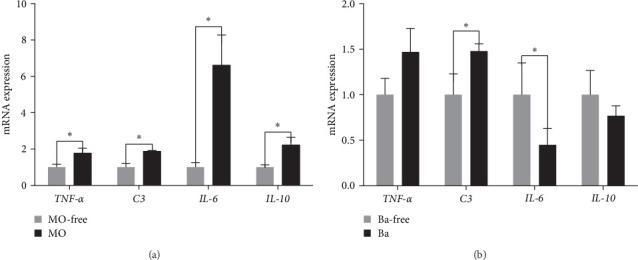
Relative gene expression of tumor necrosis factor alpha (*TNF-α*), interleukin 6 (*IL-6*), interleukin 10 (*IL-10*), complement C3 (*C3*) of Nile tilapia fed MO (a) or/and Ba (b) for 56 days. Values are expressed as mean ± SEM from quadricate groups. Bars with different letters are significantly different from those of control group (*P* < 0.05).

The mRNA expression values of *IL-6, IL-10, TNF-α*, and *C3* in the spleen of Nile tilapia fed different diets for 56 days are presented in Figure 1. There were significant upregulations of the mRNA expression values of *IL-6, IL-10, TNF-α*, and *C3* with MO supplementation in comparison with no MO supplementation (Figure 1(a), *P* < 0.05). However, there was a significant upregulation in the expression values of *C3* and downregulation in the expression values of *IL-6* with Ba supplementation compared with no Ba supplementation (Figure 1(b), *P* < 0.05).

### 3.7. Gut Microbiota and Water Microbiota Analysis

#### 3.7.1. Alpha Diversity Index

**Figure 2:**
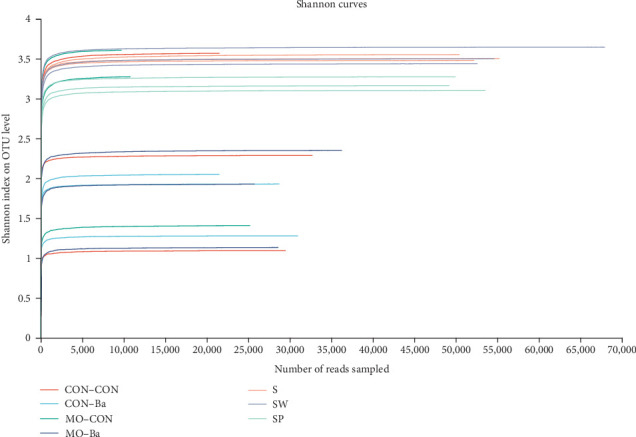
Rarefaction curves of Shannon index. *Notes*. CON–CON stands for the no treatment diet; CON–Ba stands for 0% MO and 1.0 × 10^7^ CFU/g Ba; MO–CON stands for 10% MO and 0 CFU/g Ba; MO–Ba stands for 10% MO and 1.0 × 10^7^ CFU/g Ba. S represents the pond water at 0 day; SW is the pond water of group CON–CON and group MO–CON; SP is the pond water of group CON–Ba and group MO–Ba.

**Figure 3:**
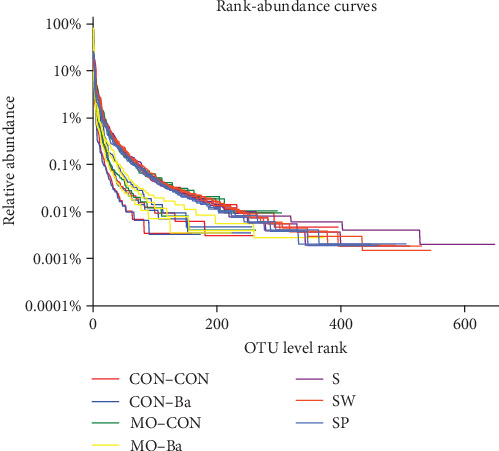
Rank-abundance curves. *Notes*. CON–CON stands for the no treatment diet; CON–Ba stands for 0% MO and 1.0 × 10^7^ CFU/g Ba; MO–CON stands for 10% MO and 0 CFU/g Ba; MO–Ba stands for 10% MO and 1.0 × 10^7^ CFU/g Ba. S represents the pond water at 0 day; SW is the pond water of group CON–CON and group MO–CON; SP is the pond water of group CON–Ba and group MO–Ba.

Data analysis of 21 samples was completed, and a total of 1,072,622 optimized sequences were obtained. Based on 97% sequence identity, 1,284 OTUs were obtained and used in subsequent analyses. A Shannon curve was established by using the number of sequences as the abscissa and the Shannon index at the OTU level as the ordinate (Figure 2). All curves reached a plateau, showing that increasing the number of brochures would not result in the appearance of new OTUs. A rank-abundance curve was constructed by using the number of OTUs and the relative percentage of species at the OTU level (Figure 3). All curves tended to flatten, indicating that the number of sequence analyses in this experiment was reasonable and might sufficiently represent the microbial diversity in each sample.

Significant differences were found between microbiota alpha diversity indices of intestinal and water samples (Table 8, Kruskal–Wallis *H* test, *P* < 0.05). The Ace index and Chao index of water samples were significantly higher than those of the intestinal samples, indicating that the species richness in water is higher than that in the intestines of Nile tilapia. The Shannon index of the water samples was higher than that of the intestinal samples, while the Simpson index was lower than that of the intestinal samples, reflecting a higher species diversity in the water than in the Nile tilapia intestine. The rank-abundance curve (Figure 3) also showed that the curve of intestinal samples exhibited a steeper decline compared to that of the water samples, indicating a higher proportion of dominant bacteria in the intestine. The coverage estimate was above 99.00% for all the samples, indicating that most of the microbial species had been detected. The *α* diversity indices differed significantly in each water group (Table 8). The SP treatment significantly lowered the Shannon and Simpson indices.

#### 3.7.2. Composition Analysis

**Figure 4:**
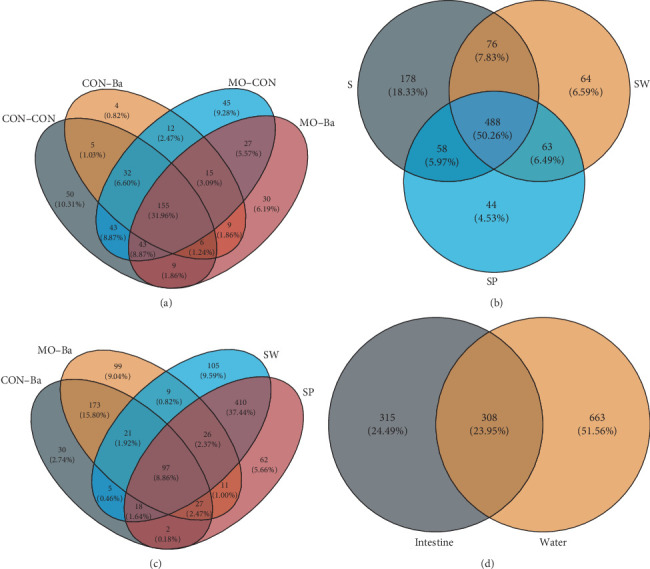
Venn diagrams of the OTU of microflora in the intestinal of Nile tilapia and water. *Notes*. The numbers in the figures represent the OTU number and percent in different groups: (a) the Venn diagram of intestinal microflora in Nile tilapia feeding different types of diets; (b) the Venn diagram of water microflora in three different ponds; (c) the Venn diagram of water microflora of SW and SP and intestinal microflora of CON–Ba and MO–Ba; (d) the Venn diagram of water microflora and intestinal microflora.

A multi-Venn diagram (Figure 4) was used to identify the OTUs shared and unique among groups. The OTUs shared by the four groups of Nile tilapia intestinal samples accounted for 12.42% of the total OTUs. However, the OTUs shared by MO supplementation in the intestinal samples accounted for 40.04% of the total OTUs. The OTUs shared by intestinal samples after Ba supplementation accounted for 38.53% of the total OTUs (Figure 4(a)). The specific OTUs of intestinal samples with MO supplementation in diets were 102, accounting for 12.61% of all samples. The specific OTUs of intestinal samples with Ba supplementation in diets were 43, accounting for 5.45% of the total number of OTUs (Figure 4(b)). Regarding water samples, the specific OTUs of Group S accounted for 22.25% of the total OTUs, while Group SW accounted for 9.26% and Group SP accounted for 6.74% (Figure 4(c)). The shared OTUs among Groups S, SW, and SP were 61.00%, 70.62%, and 74.73%, respectively. The number of unique OTUs in the water samples was 663, accounting for 42.59% of the total OTUs in the water and intestinal samples. The number of unique OTUs in intestinal samples was 315, accounting for 19.76% of the total OTUs. The number of shared OTUs between water and intestinal samples was 308, accounting for 31.72% and 49.44% of each group's respective OTUs.

**Figure 5:**
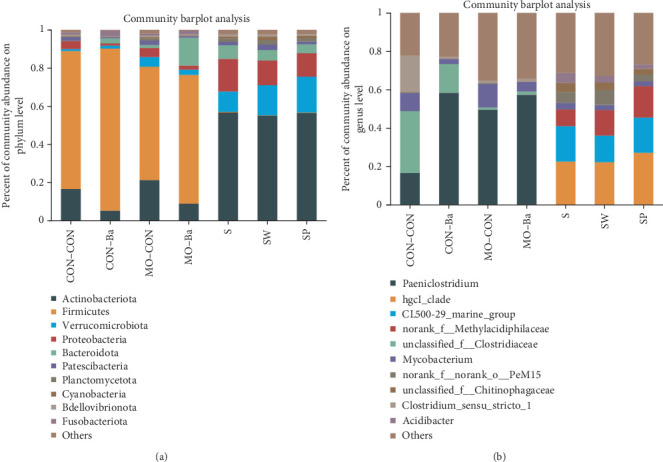
The community bar pie represents the average percentage of phylum (a) and genes (b) of microflora in intestinal and water. *Notes*. Values in the table show the relative abundance of bacteria. CON–CON stands for the no treatment diet; CON–Ba stands for 0% MO and 1.0 × 10^7^ CFU/g Ba; MO–CON stands for 10% MO and 0 CFU/g Ba; MO–Ba stands for 10% MO and 1.0 × 10^7^ CFU/g Ba. S represents the pond water at 0 day; SW is the pond water of group CON–CON and group MO–CON; SP is the pond water of group CON–Ba and group MO–Ba.

**Figure 6:**
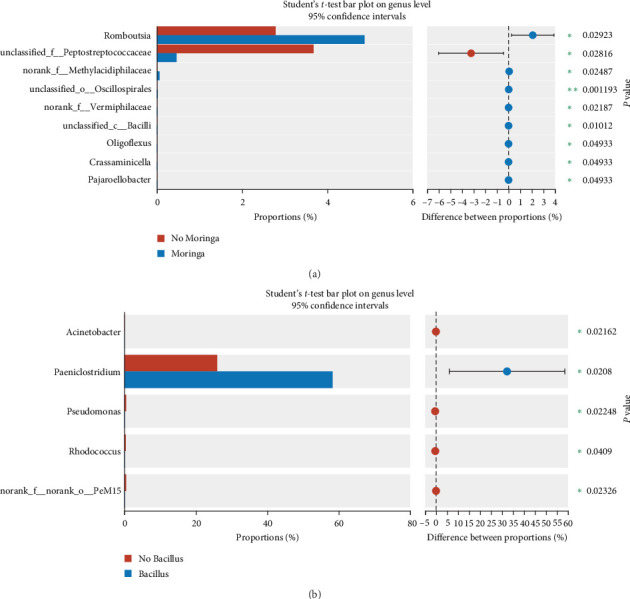
Wilcoxon rank-sum test bar plot on genus level. *Notes*. The numbers in the figures represent the *P* value of test: (a) the bar plot of intestinal microflora in Nile tilapia feeding MO of diets; (b) the bar plot of intestinal microflora in Nile tilapia feeding Ba of diets.

**Figure 7:**
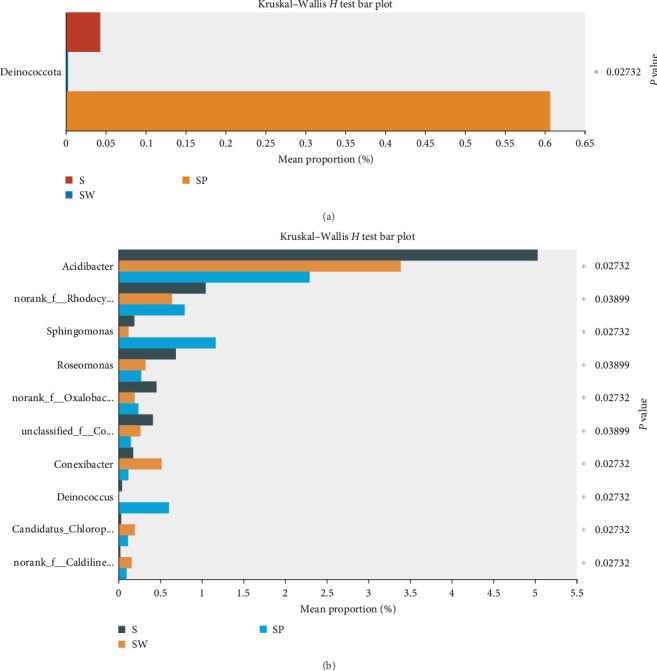
Wilcoxon rank-sum test bar plot on phylum (a) and genus (b) level of water. *Notes*. The numbers in the figures represent the *P* value of test; S represents the pond water at 0 day; SW is the pond water of group CON–CON and group MO–CON; SP is the pond water of group CON–Ba and group MO–Ba.

**Figure 8:**
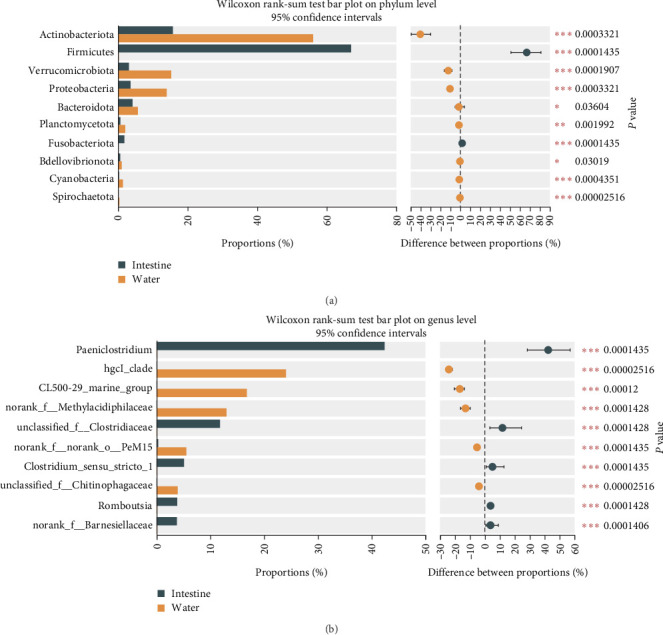
Wilcoxon rank-sum test bar plot on phylum level (a) and genus level (b) of water and microflora. *Notes*. The numbers in figures represent the *P* value of test; the bar plot of intestinal microflora and water microflora.

The intestinal microbiota can be classified into 18 phyla and 260 genera. The water microbiota can be classified into 30 phyla and 409 genera. *Firmicutes, Actinobacteriota*, and *Bacteroidota* are the dominant phyla in all intestinal groups, while the dominant phyla in the water microbiota were *Actinobacteriota, Verrucomicrobiota*, and *Proteobacteria* (ranked by abundance, with the top three accounting for over 80% of the total abundance, Figure 5(a)). In Groups S, SW, and SP, the dominant phyla of microbiota were essentially the same, with *Verrucomicrobiota* accounting for the highest proportion in Group SP (19.05%), followed by Group SW (16.14%) and Group S (10.67%). There was no significant difference in the abundance of phyla in the intestinal microbiota (*P* > 0.05). However, in the water samples, there was a significant difference in the abundance of *Deinococcota* (*P* < 0.05), with the highest abundance observed in the SP group (Figure 5(a)). To further analyze the composition of the microbiota, further analyses were carried out at the genus level (Figure 5(b)). As shown by the results, hgcl_clade, CL500-29_marine_group, and norank_f__*Methylacidiphilaceae* were the dominant genera in the water microbiota, with abundances ranging from 49.82% to 61.87%. The dominant bacterial genera in the intestinal microbiota were *Paeniclostridium*, unclassified_f__*Clostridiaceae*, and *Mycobacterium*. Although these dominant genera are similar in composition, there are great differences in abundance. The abundance of *Paeniclosidium* in the CON–CON was only 14.97%, while that in the other three groups was 58.37%, 49.57%, and 57.26%. The abundance ratios of unclassified_f__*Clostridiaceae* in each treatment microbiota were 32.23%, 14.95%, 1.08%, and 1.77%. We analyzed the effects of MO and Ba supplementation on the abundance of bacteria at the phylum and genus levels (Figure 6, the Kruskal–Wallis *H* test). Dietary MO diets markedly increased the abundance of *Romboutsia* in the microbiota of the intestine (*P* < 0.05). *Peptostreptococcaceae* was detected in all intestinal samples; however, the addition of MO significantly reduced the abundance of *Peptostreptococcaceae (*P* < 0.05*). Furthermore, supplementation with Ba can increase the abundance of *Paeniclosidium* in the intestines of Nile tilapia, and the abundance of *Acinetobacter* also significantly decreased (*P* < 0.05). Additionally, the proportion of the abundance of *Mycobacterium* in the intestinal microbiota of the Ba groups was 10% lower than that of the non-Ba group. Additionally, the abundance of *Acidibacter* in water samples significantly decreased after feeding diets compared with the control group (S) (Figure 7). Additionally, after feeding the diet supplemented with Ba, the abundance of *Sphingomonas* in Group SP significantly increased, while the abundance of *Roseomonas* significantly decreased (*P* < 0.05). In comparison with intestinal samples of Nile tilapia, the abundance of *Firmicutes* in water microbiota was significantly lower than that in intestinal microbiota (Figure 8). The abundance of *Paeniclosidium* was also significantly lower than that in the intestinal microbiota (Figure 8).

**Figure 9:**
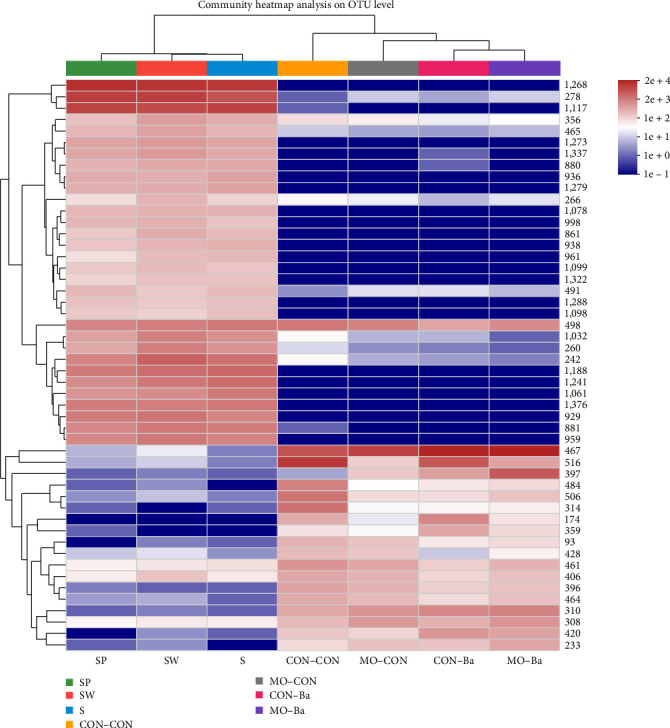
Heatmap analysis of the bacteria community on OTU level. *Notes*. CON–CON stands for the no treatment diet; CON–Ba stands for 0% MO and 1.0 × 10^7^ CFU/g Ba; MO–CON stands for 10% MO and 0 CFU/g Ba; MO–Ba stands for 10% MO and 1.0 × 10^7^ CFU/g Ba. S represents the pond water at 0 day; SW is the pond water of group CON–CON and group MO–CON; SP is the pond water of group CON–Ba and group MO–Ba.

**Figure 10:**
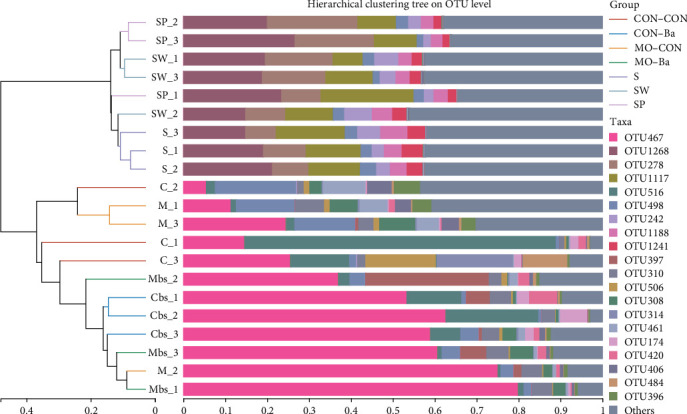
The microbial community heatmap (at phylum level) of microflora in intestinal and water. *Notes*. CON–CON stands for the no treatment diet; CON–Ba stands for 0% MO and 1.0 × 10^7^ CFU/g Ba; MO–CON stands for 10% MO and 0 CFU/g Ba; MO–Ba stands for 10% MO and 1.0 × 10^7^ CFU/g Ba. S represents the pond water at 0 day; SW is the pond water of group CON–CON and group MO–CON; SP is the pond water of group CON–Ba and group MO–Ba.

**Figure 11:**
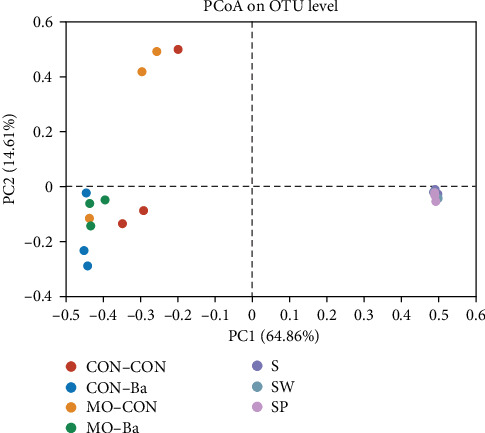
The PCoA on OTU level of microflora in intestinal and water. *Notes*. Comparison of intestinal and water samples among all groups using PCoA analysis. The distance between points shows the similarity of microbiotic. CON–CON stands for the no treatment diet; CON–Ba stands for 0% MO and 1.0 × 10^7^ CFU/g Ba; MO–CON stands for 10% MO and 0 CFU/g Ba; MO–Ba stands for 10% MO and 1.0 × 10^7^ CFU/g Ba. S represents the pond water at 0 day; SW is the pond water of group CON–CON and group MO–CON; SP is the pond water of group CON–Ba and group MO–Ba.

As shown in Figure 9, the intestinal microbiota of the CON–Ba and MO–Ba are clustered together in the community heatmap analysis, and there was an obvious difference in the combination of microbial communities (color) between water samples and intestinal samples. As shown in the hierarchical clustering tree (Figure 10), the intestinal samples treated with Ba supplementation in the diet are also clustered in the same branch. The PCoA showed that the bacterial community in water was clustered in the fourth quadrant (Figure 11), while the intestinal samples were mainly clustered in the second and third quadrants; among them, the CON–Ba and MO–Ba were clustered in the third quadrant.

#### 3.7.3. Functional Prediction Analysis of Microbiota

**Figure 12:**
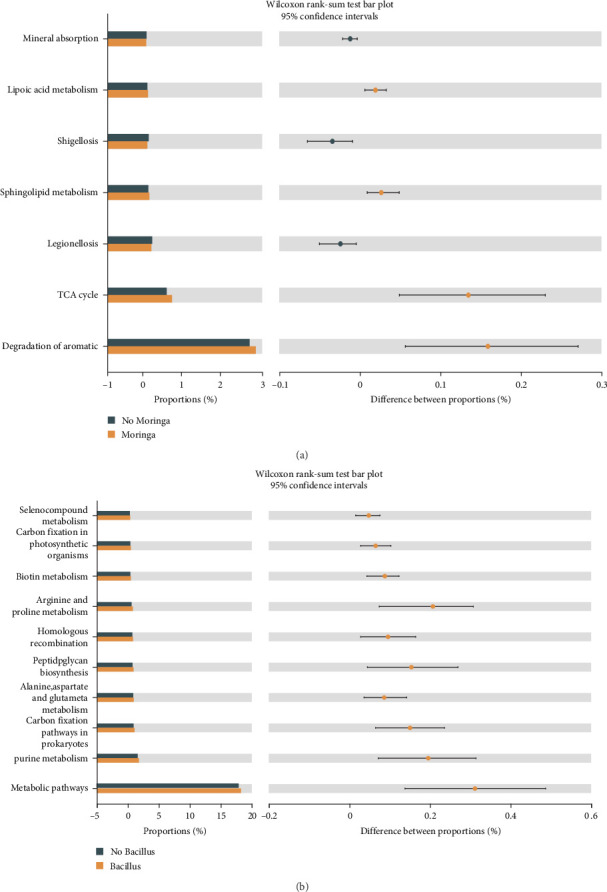
Wilcoxon rank-sum test bar plot on the predicted pathway of intestinal samples with and without MO (a) and Ba (b).

As shown in Table 9, in each treatment group, a total of 357 metabolic pathways were identified, including 23 pathways related to cellular processes, 27 pathways related to environmental information processing, 69 pathways related to human disease, 156 pathways related to metabolism and 64 pathways related to organismal systems. MO supplementation increased the abundance of lipid metabolism, TCA cycle, degradation of aromatic compounds, and sphingolipid metabolism pathways in the intestines of Nile tilapia (Figure 12). The comparison between dietary Ba supplementation and no Ba supplementation revealed 58 different pathways. The top 20 metabolic pathways were selected based on abundance. After feeding with Ba, the abundance of metabolic pathways, alanine, aspartate, and glutamate metabolism purine metabolism, carbon fixation pathways in prokaryotes, biotin metabolism, peptidoglycan biosynthesis, homologous recombination, arginine, and proline metabolism, carbon fixation in photosynthetic organisms and seleno-compound metabolism in the intestinal microbiota of Nile tilapia were increased (Figure 12).

## 4. Discussion

In this experiment, FBW (g), WGR (%), and SGR (%/day) of Nile tilapia were significantly lower when fed diets with 10% *M. oleifera* added compared with diets without *M. oleifera*. This is contrary to previous experimental results. Studies have reported that supplementation with 10% *M. oleifera* had no negative effect on the growth performance of Nile tilapia [27]. A study also found that 12% of *M. oleifera* added to Nile tilapia diets can significantly improve growth performance [28]. However, unlike in this experiment, these two experimental fish were reared in the laboratory conditions, and the total feed intake was limited. The fish in this experiment were reared in the outdoor pond and fed to apparent satiation. During feeding, it was observed that the fish showed a low level of initiative towards eating the feed supplemented with *M. oleifera* (MO–CON, MO–Ba). We hypothesize that this can be attributed to the characteristic odor of *M. oleifera*, which may result in poor palatability of the feed [29]. The taste reception is important for the choice of food selected by fish. The palatability of animals (such as plankton) is higher than that of plants extract [30]. Besides, replacing fishmeal with plant-based ingredients increases the crude fiber content of the feed. Fish have few enzymes to digest high-fiber materials, which reduces their ability to obtain nutrients from plant-based diets [31]. Statistically significant differences were found on Day 56 between the *M. oleifera* diets, which may be the main reason for the lower growth performance of the MO group. Although *M. oleifera* may not further enhance the growth performance of fish, in this experiment, adding 10% *M. oleifera* to the diet significantly reduced the FCR and improved the PCR. This indicates that *M. oleifera* added to feed can save protein costs, but it is necessary to address the problem of poor palatability of *M. oleifera* in fish. Djissou et al. [32]. examined the effect of completely replacing *M. oleifera* leaves with fish meal on Nile tilapia. They found that the SGR was lower than that of the untreated group after 6 weeks of feeding. With increasing concentrations of *M. oleifera* in the diet, the weight gain of *C. gariepinus* decreased [33]. Due to the difficulty of absorbing structural carbohydrates in the intestines and stomach of animals, *M. oleifera* has a high content of crude fiber. Elevated levels of crude fiber may increase the energy loss for digestion, thereby causing a negative effect on growth performance. To solve the problem of poor palatability of plant-source feed, according to current research, the reduction of antinutritional factors in feeds through fermentation is a prospective way to enhance the quality of woody feeds [34–36]. The finding of the current study indicated that supplementation with 1 × 10^7^ CFU/g Ba improved the growth performance of Nile tilapia. Previous studies using *B. amyloliquefaciens* (isolated from the *Tachysurus fulvidraco*) also observed an enhancement in growth performance [37]. *B. amyloliquefaciens* and *B. pumilus* added to the feed improved the growth of striped catfish [38]. One of the objectives of this experiment was to explore the synergy between *M. oleifera* and *B. amyloliquefaciens* supplementation. Although *M. oleifera* and *B. amyloliquefaciens* did not result in a significant synergistic effect on growth improvement; the improvement in growth was greater with *B. amyloliquefaciens* supplementation in the *M. oleifera* diet (WGR with MO–Ba treatment: 349.42%, WGR with MO–CON treatment: 333.68%) than in the control treatment (WGR with CON–Ba treatment: 377.61%, WGR with CON–CON treatment: 368.78%).

In aquaculture, the condition factor (CF) is one criterion used to assess fish growth [39]. Although the CF cannot represent the overall growth performance of fish, in this study, *M. oleifera* and *B. amyloliquefaciens* supplementation significantly enhanced the VSI and GSI of Nile tilapia, and these two additions had an interaction with VSI and GSI. The MO–Ba group exhibited the highest increase in morphological indices. Dietary supplementation with *M. oleifera* and *B. amyloliquefaciens* can promote the development of the visceral and intestines of Nile tilapia, as well as improve the CF. This may indicate that *M. oleifera* and *B. amyloliquefaciens* can promote visceral development in Nile tilapia.

The whole-body crude fat content of Nile tilapia was significantly decreased as dietary MOL levels increased [40]. A previous study showed that *M. oleifera* can inhibit the accumulation of lipids in the body in a ZOT [41]. It seems that *M. oleifera* reduces the whole-body fat of animals. Dietary supplementation with *B. amyloliquefaciens* improved the whole-body crude fat content of Nile tilapia. An earlier study reported that the crude protein requirement in Nile tilapia feed should be approximately 33% [42]. In fact, the initial weight of fish and the environment of aquaculture should be taken into consideration. However, in our experiment, the diet of MO–CON contained 34.5% crude protein, and MO–Ba contained 34.6% crude protein, which is both lower than CON–CON (35.4%) and CON–Ba (35.5%). Higher crude protein content was observed in the whole body of Nile tilapia after feeding with *M. oleifera*, which indicates that *M. oleifera* has a positive effect on protein accumulation in Nile tilapia. However, further studies on the composition of *Moringa* (amino acids carrying nutrient factors) are needed to determine the most appropriate level of *M. oleifera* to be added to animals.

A combination of protease, lipase, and amylase is required for fish digestion. In an vitro experimental assessment, the extract of *M. oleifera* was reported to inhibit the activity of *α*-amylase and lipase [43]. Low levels of digestive enzyme activity cannot stimulate the hydrolysis of diets, which may make it difficult to absorb the nutrients in the feed. However, *B. amyloliquefaciens* in the diet significantly improved the amylase and protease activity in the intestine of *Rhynchocypros lagowski* [44]. In a previous study, *M. oleifera* had no significant impact on the activity of digestive enzymes, while diets supplemented with *B. amyloliquefaciens* had a higher activity of *α*-amylase than diets without *B. amyloliquefaciens*. Antinutritional factors in plant protein feed, such as trypsin inhibitors, tannins, and saponins, inhibit protease activity in fish. In this experiment, *M. oleifera* had a negative effect on the activity of protease in the stomach, while *B. amyloliquefaciens* addition significantly improved the activity of proteases in the stomach. A marked improvement in the activity of protease was also observed in *Apostichopus japonicus* fed *Bacillus baekryungensis* [45]. Probiotics can promote growth by increasing the amount of endogenous digestive enzymes secreted by fish [46]. As in our present study, *B. amyloliquefaciens* can improve the reactivity of digestive enzymes. We concluded that in the scenario where plant protein feed may inhibit the activity of digestive enzymes in aquatic animals, *B. amyloliquefaciens* has a positive effect on the digestion of aquatic animals.

The spleen is a major immune organ that engages in hematopoietic functions. *M. oleifera* effectively improves the immunity of animals [47–49]. In the present study, the content of immune factors (*IL-6, TNF-α, C3*) in the spleen was significantly increased in Nile tilapia after feeding with *M. oleifera* feed as determined by ELISA. Similarly, the mRNA expression of *IL-6, IL-10, TNF-α*, and *C3* was significantly upregulated in the spleen tissue of Nile tilapia in treated with *M. oleifera*. Supplementation with *M. oleifera* in the feed resulted in a significant increase in *TNF-α* expression in Nile tilapia [50], similar to our findings. Findings were found in Nile tilapia, with the addition of *M. oleifera* to the feed can increase the mRNA expression of *TNF-α* [51]. It was reported that *TNF-α* can be a proinflammatory factor in the immunity of Nile tilapia [52], as well as a cytokine related to lipid metabolism [53]. In addition, *TNF-α* can induce lipolysis and promote inflammation [54]. In our prevention study, the content of *TNF-α* in the spleen and the mRNA expression of *TNF-α* were significantly increased after feeding with *M. oleifera*, which is consistent with the trend that *M. oleifera* can exert a hypolipidaemic function [55]. *IL-6* produced by macrophages plays a role in the immune response and hematopoietic regulation and is an important inflammatory factor [56]. *IL-6* can participate in the defense against bacteria and promote the production of antibodies [57]. Notably, *IL-6* is a novel regulator of lipolysis that has been shown to accelerate lipid degradation in sheep [58]. In this experiment, *M. oleifera* significantly increased the expression of *TNF-α* and *IL-6* gene in the spleen compared with non-*M. oleifera* treatment and the whole-body crude fat content was also significantly reduced. We speculate that this may be related to the antiadipogenic properties of *M. oleifera*. *IL-10* is involved in the defense against bacteria and promotes the production of antibodies such as *IL-6* [59]. Additionally, *IL-10* was found to have anti-inflammatory activity [60]. Anti-inflammatory cytokines are an effective tool that can be used to eliminate inflammation to restore the body to normal physiological levels after inflammatory cytokines destroy the pathogen. The balance between inflammatory and anti-inflammatory cytokines is a key factor in maintaining the body's normal immune status stability and normal physiological activity. In our study, the mRNA expression of *IL-6* and *TNF-α* in the spleen was significantly upregulated after feeding with MO–CON and MO–Ba; however, the mRNA expression of *IL-10* was also upregulated. In vitro antifungal activity against *Saprolegnia* spp. experiment [61], treatment with *M. oleifera* (MS-AgNPs) upregulated *IL-10* and *TNF-α* mRNA expression in the head kidney of *Oreochromis niloticus*. This may be related to the fact that *Moringa* exerts its own unique disease-resistant functions. C3 is effective in the innate immune response to the host's immune defense mechanisms. After bacterial infection of the organism, *C3* can reduce the inflammatory response and pathological damage to tissues [62]. In some fish species, when the mRNA expression of *C3* decreases in the body, the complement activity of the serum will decrease, and the abundance of pathogenic bacteria will increase [63]. Both *M. oleifera* and *B. amyloliquefaciens* supplementation significantly increased the mRNA expression of *C3* in the spleen of Nile tilapia, and their combination could show a greater effect on the mRNA expression of *C3*. This combination could possibly be expected to improve fish resistance to pathogenic bacteria and enhance fish complement activation.

The intestinal microbiota has a close relationship with animal digestion, nutrient metabolism, and the immune system. In addition to affecting the growth of animals, intestinal microflora can help analyze the mechanism of disease resistance [64, 65]. The structure, composition, and diversity of the intestinal microbiota of animals are related to the habitat, feed consumed, and genetics of the species. However, apart from studies on the effect of different feed types on the intestinal microflora, research on the microbiota between the environment and fish remains underdeveloped, especially studies on the effect of feed-containing probiotics. In this study, different diets were used to evaluate the effect on the intestinal microbiota and water microbiota to further understand the relationship between the environment and fish. Current studies on microflora have focused on the intestines of animals, but there are also some studies that were different. Turcihan et al. [66] studied the changes in dominant bacteria in cultured water by feeding different types of diets. Lv et al. [67] analyzed the effect of microbiota on the survival of marble gobies after skin abrasion. Microbiota is influenced by different factors, including culture mode [68], feeding methods [69, 70], feed type [71], growth stage of fish [72], etc. However, few studies have been conducted on the linkage between intestinal and environmental microbiota. It has been found that the diversity and homogeneity of intestinal microbiota can be improved by feeding probiotics [73]. It should be noted that there have been experiments in which the homogeneity of intestinal microbiota was significantly reduced after the addition of Bacillus to the feed [74]. The diversity of microflora in fish is an indicator of growth development, but an index of diversity that is too high or too low will have a negative impact on fish. In this experiment, the four different diets had no significant effect on the alpha diversity. To explore the diversity of intestinal microbiota related to fish health, the complex interactions between hosts and microorganisms need to be further investigated. By comparing the alpha diversity index of the intestinal and water samples, we found that the diversity and abundance of microbial communities in water were higher than those in the intestine, which is similar to a previous study [75]. This may be attributed to the fact that the intestine can adapt to changes in the external environment and maintain a stable microflora. In aquaculture, it has been shown that the gut microbiota can be altered by feeding different diets [76–78]. In this experiment, both *M. oleifera* and *B. amyloliquefaciens* altered the similarity of Nile tilapia intestinal microbiota, but this effect did not differ significantly. However, considering the number of respective specific OTUs, *M. oleifera* seemed to more easily alter the specificity of microbiota than *B. amyloliquefaciens*. After 8 weeks of feeding, the treatment water (SW and SP) microbiota showed a decrease in the number of specific OTUs compared to the control water (S), indicating that the microbiota in water was affected by feed, as evidenced by a reduction in the specificity and an increase in the similarity of the microflora composition. The number of unique OTUs shows that the specificity of the water microbiota is higher than that of the intestinal microbiota, and the common OTUs are lower. Similar to the alpha diversity results, the water microbiota has a higher diversity and abundance than the intestinal microbiota. This may suggest that the water microbiota is more susceptible to climatic or anthropogenic. Taxonomically, *B. amyloliquefaciens* belongs to the class *Bacilli* and phylum *Firmicutes*. In the present experiment, after feeding with *B. amyloliquefaciens*, the abundance of *Firmicutes* in intestinal samples increased, while the abundance of *Proteobacteria* was reduced. The same result was also reported on *Scophthalmus maximus* [79]. *B. amyloliquefaciens* can affect the abundance of *Firmicutes* in the intestinal microbiota of Nile tilapia. However, it is necessary to conduct further research on the transcription and translation of *B. amyloliquefaciens* in the intestine to determine whether *B. amyloliquefaciens* can stably colonize the intestine. At the genus level, *Peptostreptococcaceae* was detected in all intestinal samples. In fact, *Peptostreptococcaceae* infections have become a major problem in tilapia farming and contribute to enormous damage [80]. The abundance of *Peptostreptococcaceae* in the MO–CON and MO–Ba groups was significantly lower than those in the CON–CON and CON–Ba groups. This may be related to the antibacterial activity of *M. oleifera* [81]. The reduction in *Peptostreptococcaceae* is favorable due to the danger it poses to Nile tilapia farming. The abundance of *Romboutsia* in the MO–CON and MO–Ba groups was significantly higher than that in the CON–CON and CON–Ba groups. *Romboutsia* could be helpful for butyric acid production [82]. Butyric acid plays a significant role in promoting gut health and immune system regulation. In this experiment, we also found that the supplementation with *M. oleifera* reduced the whole-body crude fat of Nile tilapia. Whether there is a relevant relationship between *M. oleifera* and butyric acid need further study. At the phylum level, the abundance of *Deinococcota* in the SP group was significantly higher than that in the other groups (S, SW), while *Deinococcus-Thermus* has been confirmed to possess strong resistance against environmental hazards, indicating that *B. amyloliquefaciens* has the potential to promote beneficial bacteria in the water environment by feeding in the process of aquaculture [83]. At the genus level, the abundance of *Sphingomonas* in the SP group was significantly higher than that in the other groups. *Sphingomonas* participates in the degradation of organic metal compounds [84], but in our study, we did not perform a comprehensive measurement of the biochemical indicators of water, so the mechanism of *Sphingomonas* for environmental remediation is still unclear. *Roseomonas* is a potentially pathogenic bacterium that has been confirmed to be a genus of bacteria that can cause immune system suppression in humans [85]. Although great harm from *Roseomonas* has not been found in aquaculture, we found that the abundance of *Roseomonas* in group SP was significantly lower than in the S and SW groups. After feeding with the diets containing *B. amyloliquefaciens*, the abundance of beneficial bacteria in water (in the SP group) was significantly increased, while the abundance of potentially pathogenic bacteria was decreased. Wang et al. [86] also assessed the effect of *Bacillus* sp. on the gut microbiota of Nile tilapia (as an additive or water treatment), and they found that both methods could alter the microbiota of the intestine. However, as a feed additive, it was more effective in improving the intestinal microbiota status. The abundance of *Firmicutes* in the microbiota of water samples was significantly lower than that in intestinal samples in this experiment, and *Bacillus* was almost undetectable in each water sample. This may imply that after feeding with a diet containing *B. amyloliquefaciens*, the strain can better colonize the gut than water. The heatmap analysis of the bacteria community presents the microbial composition and abundance of different bacterial species. It is usually clustered based on the similarity of varied species and reflects the similarity and differences in the composition of microbial communities in different groups through the color changes in the Figure 9. The heatmap analysis showed that the CON–Ba and MO–Ba groups were clustered in the same branch. We used a heatmap analysis to further compare the water and intestinal samples, and cluster analysis showed distinct patterns of microbiota compositions between water and intestinal microbiota. In addition, the PCoA analysis also found a similar trend to that of heatmap analysis. The predominant genera in Nile tilapia were *Firmicutes, Actinobacteria*, and *Proteobacteria* [87]. Our study showed that in the intestines of the four treatment groups, there was a high abundance ratio of *Firmicutes* and *Actinobacteria* (CON–CON: 88.91%; CON–Ba: 90.2%; MO–CON: 80.73%; MO–Ba: 76.47%). These results indicate that this species of bacteria plays a significant role in the intestinal microbiota of Nile tilapia, possibly in nutrient metabolism or feed absorption. The microbiota of the same fish species may be specific. For example, the *black sea bream* collected from the same area had different microbiota, and samples from the same group did not cluster together effectively in PCoA analysis [88]. However, the habitat and feed cannot entirely influence the changes in the microbiota, as this is more dependent on the host's specificity and the colonization ability of bacteria. Further analysis of the KEGG pathways for microbial metabolism predicted by PICRUSt1 showed that three pathways (lipoic acid metabolism, sphingolipid metabolism, and TCA cycle) were more abundant in the *M. oleifera* groups. These results indicated that *M. oleifera* influenced intestinal bacteria metabolism functions, and these functions are related to lipid metabolism, which may be relevant to the regulation of lipid metabolism by *M. oleifera* [89]. Understanding the metabolic functions and nutrient absorption of intestinal microbiota after feeding with *M. oleifera* may potentially reveal the pathways by which *M. oleifera* affects the involvement of microbiota in lipid metabolism. Analyzing the 20 most highly different predicted pathways of intestinal bacteria in Nile tilapia after feeding the diet with *B. amyloliquefaciens*, we found that 9 pathways are related to metabolism, including energy metabolism, amino acid metabolism, nucleotide metabolism, metabolism, metabolism of cofactors, and vitamins and glycan biosynthesis. Further analyzing the pathway related to amino acid metabolism (aspartate and glutamate metabolism, alanine, arginine, and proline metabolism) showed that the abundance of the amino acid metabolism pathway was higher in (CON–Ba and MO–Ba) groups than in the groups without Ba. The abundance of amino acid metabolism pathways in the microbiota of the intestine is related to changes in nutritional status [90], which can explant why the weight gain of Nile tilapia fed with *B. amyloliquefaciens* was better than that not fed with *B. amyloliquefaciens*.

## 5. Conclusion

In conclusion, dietary uses of *M. oleifera* and *B. amyloliquefaciens* showed different effects on the growth performance of Nile tilapia. However, dietary use of 1 × 10^7^ CFU/g *B. amyloliquefaciens* enhanced weight gain with the largest improvement in CON–Ba, while the 10% *M. oleifera* used in diets improved the feed utilization. In addition, both *M. oleifera* and *B. amyloliquefaciens* improved the immune response and related gene expression, whereas *B. amyloliquefaciens* enhanced the activity of digestive enzymes in Nile tilapia. The results of this experiment showed that there were significant differences in the composition and structure of the microflora of the farming water and the microbiota in the intestine of Nile tilapia. Based on the results of the two-way ANOVA analysis, no interaction effect was found between *M. oleifera* and *B. amyloliquefaciens* in terms of growth performance on Nile tilapia. However, we believe that adding *B. amyloliquefaciens* to the feed can promote the growth of Nile tilapia. Although *M. oleifera* can save feed costs and improve the immunity of fish, and further research is necessary to address the issue of poor palatability of *M. oleifera* feed. So, this research provides evidences about the positive effects of *B. amyloliquefaciens* improving the growth performance of Nile tilapia. The supplementation of *M. oleifera* in the Nile tilapia diet helps to formulate healthy and cost-effective fish feed.

## Figures and Tables

**Table 1:** Formulation and proximate composition of the experimental diets (%).

Ingredients (%)	CON–CON	CON–Ba	MO–CON	MO–Ba
*M. oleifera* powder	0.0	0.0	10.0	10.0
*B*. *amyloliquefaciens*	—	+	—	+
Soybean meal	37.0	37.0	37.0	37.0
Rapeseed meal	20.0	20.0	20.0	20.0
Wheat flour	25.8	25.8	23.4	23.4
DDGS	8.0	8.0	0.0	0.0
Fish meal powder	2.0	2.0	2.0	2.0
Soybean oil (≥98%)	2.6	2.6	3.0	3.0
Perilla seed oil	0.8	0.8	0.8	0.8
Ca(H_2_PO_4_)_2_	2.0	2.0	2.0	2.0
Multivitamins*⁣*^*∗*^^a^	0.5	0.5	0.5	0.5
Vitamin C	0.2	0.2	0.2	0.2
Multiminerals*⁣*^*∗*^^b^	0.5	0.5	0.5	0.5
Methionine (≥98%)	0.4	0.4	0.4	0.4
Choline chloride	0.2	0.2	0.2	0.2
Total	100.0	100.0	90.0	90.0
Proximate composition of diets (% dry matter)				
Moisture	5.1	5.0	4.6	4.8
Crude protein	35.4	35.5	34.5	34.6
Crude lipid	5.9	5.9	6.0	6.0
Crude ash	6.9	7.1	7.7	7.6
Gross energy (kJ/g)	18.4	18.4	18.5	18.4

*⁣* ^*∗*^Multivitamins and multiminerals were supplied by Guangzhou Heng Xing Aquatic Technology Company, China. ^a^Multivitamins: vitamin C (800 mg), vitamin A (8,000,000 IU), thiamine (700 mg), riboflavin (3,500 mg), pyridoxine (1,000 mg), cyanocobalamin (7 mg), vitamin D3 (2,000,000 IU), *α*-tocopherol acetate (7,000 mg), vitamin K3 (1,500 mg), biotin (50 mg), folic acid (700 mg), niacin (20,000 mg), and pantothenic acid (7,000 mg). ^b^Multiminerals: iron (20 g), zinc (40 g), copper (2.7 g), iodine (0.34 g), cobalt (70 mg), manganese (53 g), selenium (70 mg).

**Table 2:** Primers used for RT-PCR analysis.

Target mRNA	Primer sequence (5′-3′)	Accession number
*IL-6*	F:ACAGAGGAGGCGGAGATG	XM_019350387.2
	R:GCAGTGCTTCGGGATAGAG	—

*IL-10*	F:TGGAGGGCTTCCCCGTCAG	XM_019358799.2
	R:CTGTCGGCAGAACCGTGTCC	—

*TNF-α*	F:TAGAAGGCAGCGACTCAA	NC_031985.2
	R:CCTGGCTGTAGACGAAGT	—

*C3*	F:CAGGCAGGAGGATGTATCGG	XM_005454829.4
	R:TGCCAGCGTCAAGTCTTTTCT	—

*Β-actin*	F:CAGGGAGAAGATGACCCAGA	XM_003443127.5
	R:CAGGGCATAACCCTAGTAGA	—

**Table 3:** The growth performances of Nile tilapia fed test diets for 56 days.

Treatments	CON		MO		*P* values		
	CON	Ba	CON	Ba	MO	Ba	MO × Ba
FBW (g)	164.27 ± 0.59	167.38 ± 1.62	151.95 ± 1.52	157.46 ± 1.38	<0.001 (*b/a*)	0.012 (*x/y*)	0.396
WGR (%)	368.78 ± 1.74	377.61 ± 4.70	333.68 ± 4.27	349.42 ± 3.99	<0.001 (*b/a*)	0.013 (*x/y*)	0.395
SGR (g/day %)	2.76 ± 0.01	2.79 ± 0.02	2.62 ± 0.02	2.68 ± 0.02	<0.001 (*b/a*)	0.009 (*x/y*)	0.391
FI (g/fish/day)	2.40 ± 0.15	2.46 ± 0.06	2.08 ± 0.06	2.15 ± 0.06	0.045 (*b/a*)	0.549	0.904
FCR	1.06 ± 0.11	1.10 ± 0.03	0.95 ± 0.04	0.96 ± 0.02	0.007 (*b/a*)	0.456	0.706
PER (%)	2.77 ± 0.27	2.69 ± 0.06	3.20 ± 0.11	3.14 ± 0.05	0.001 (*a/b*)	0.468	0.883
SR (%)	91.67 ± 0.83	92.50 ± 3.82	98.13 ± 0.00	93.33 ± 4.41	0.426	0.661	0.536

*Notes*: MO, *M. oleifera* supplementation; Ba, *B. amyloliquefaciens* supplementation; MO × Ba, interaction effect. Values are means and standard error of the mean (SEM). ns, no significant, *⁣*^*∗*^*P* < 0.050. Different letters indicate significant differences between the two woody types and probiotic types, such as (*a*/*b*) in parentheses corresponds to no MO addition, MO addition, and the value of *a* is less than *b*. The other set of letters (*x*/*y*) corresponds to no *B. amyloliquefaciens* addition and *B. amyloliquefaciens* addition, and the value of *x* is less than the value of *y*. IBW (g), initial body weight; FBW (g), final body weight; Survival rate (SR, %) = 100 × final number of the fish/40; Weight gain rate (WGR, %) = (FBW − IBW)/IBW; Specific growth rate (SGR, %/day) = 100 × (lnFBW − lnIBW)/days; Protein efficiency ratio (PER, %) = weight gain (g)/protein intake (g); Feed conversion ratio (FCR) = feed intake (dry weight, g)/weight gain (g); Feeding intake (FI, g/fish/day) = feed intake (dry weight, g) × 100/((IBW + FBW)/2 × days).

**Table 4:** The morphological indexes of Nile tilapia fed test diets for 56 days.

Treatments	CON		MO		*P* values		
	CON	Ba	CON	Ba	MO	Ba	MO × Ba
HSI (%)	1.35 ± 0.06	1.45 ± 0.16	1.32 ± 0.08	1.47 ± 0.08	0.758	0.312	0.794
VSI (%)	10.41 ± 0.24A	10.57 ± 0.18A	10.59 ± 0.12A	11.96 ± 0.35B	0.007 (*a*/*b*)	0.013 (*x*/*y*)	0.006
GSI (%)	7.89 ± 0.18A	7.88 ± 0.12A	8.42 ± 0.11A	9.69 ± 0.37B	0.001 (*a*/*b*)	0.042 (*x*/*y*)	0.011
CF (g/cm^3^)	3.61 ± 0.03	3.81 ± 0.04	3.96 ± 0.09	4.11 ± 0.02	<0.001 (*a*/*b*)	0.045 (*x*/*y*)	0.453

*Notes*: MO, *M. oleifera* supplementation; Ba, *B. amyloliquefaciens* supplementation; MO × Ba, interaction effect. Values are means and standard error of the mean (SEM). ns, no significant, *⁣*^*∗*^*P* < 0.05. Different letters indicate significant differences between the two MO adding types and two Ba types, such as (*a*/*b*) in parentheses corresponds to no MO addition, MO addition, and the value of *a* is less than *b*. The other set of letters (*x*/*y*) corresponds to no Ba addition and Ba addition, and the value of *x* is less than the value of *y*. The presence of different capital letters indicates a significant difference between groups. Hepatosomatic index (HSI, %) = (liver weight (g)/body weight (g)) × 100; Visceral index (VSI, %) = (visceral weight (g)/body weight (g)) × 100; Gut index (GSI, %) = (gut weight (g)/body weight (g)) × 100; Condition factor (CF, g/cm^3^) = (body weight (g)/body length^3^ (cm^3^)) × 100.

**Table 5:** The body composition of Nile tilapia fed test diets for 56 days.

Treatments	CON		MO		*P* values		
	CON	Ba	CON	Ba	MO	Ba	MO × Ba
Crude moisture (%)	74.04 ± 0.62A	72.78 ± 0.74A	72.84 ± 0.74A	73.42 ± 0.73A	0.493	0.409	0.046
Crude protein (%)	15.11 ± 0.63	15.25 ± 0.55	16.40 ± 0.11	16.14 ± 0.57	0.006 (*a*/*b*)	0.835	0.522
Crude fat (%)	6.26 ± 0.28	6.81 ± 0.84	5.18 ± 0.60	6.12 ± 0.41	0.009 (*b*/*a*)	0.023 (*x*/*y*)	0.507
Ash (%)	3.54 ± 0.09A	3.66 ± 0.16A	4.32 ± 0.26B	3.66 ± 0.29A	0.004 (*a*/*b*)	0.030 (*y*/*x*)	0.004

*Notes*: MO, *M. oleifera* supplementation; Ba, *B. amyloliquefaciens* supplementation; MO × Ba, interaction effect. Values are means and standard error of the mean (SEM). ns, no significant, *⁣*^*∗*^*P* < 0.05. Different letters indicate significant differences between the two MO adding types and two Ba types, such as (*a*/*b*) in parentheses corresponds to no MO addition, MO addition, and the value of *a* is less than *b*. The other set of letters (*x*/*y*) corresponds to no Ba addition and Ba addition, and the value of *x* is less than the value of *y*. The presence of different capital letters indicates a significant difference between groups.

**Table 6:** The digestive enzymes of Nile tilapia fed test diets for 56 days.

Treatments	CON		MO		*P* values		
	CON	Ba	CON	Ba	MO	Ba	MO × Ba
Foregut							
LPS (U/gprot)	0.78 ± 0.20	3.40 ± 0.80	2.37 ± 0.33	2.64 ± 0.49	0.465	0.022 (*x*/*y*)	0.055
AMS (U/mgprot)	7.23 ± 0.85	13.78 ± 2.54	9.05 ± 1.90	15.27 ± 2.95	0.882	0.005 (*x*/*y*)	0.543
CAS (U/mgprot)	3.65 ± 0.32	2.22 ± 0.27	2.76 ± 0.99	2.73 ± 0.41	0.745	0.233	0.249
Midgut							
LPS (U/gprot)	3.13 ± 0.64	2.00 ± 0.25	3.65 ± 0.68	2.93 ± 0.41	0.194	0.105	0.711
AMS (U/mgprot)	5.07 ± 0.26	5.49 ± 0.30	5.76 ± 0.67	5.24 ± 0.10	0.487	0.688	0.822
CAS (U/mgprot)	2.21 ± 0.30	2.71 ± 0.33	2.63 ± 0.59	2.57 ± 0.13	0.719	0.575	0.477
Hindgut							
LPS (U/gprot)	1.73 ± 0.39	2.48 ± 0.25	1.50 ± 0.03	1.87 ± 0.07	0.108	0.045 (*x*/*y*)	0.436
AMS (U/mgprot)	4.78 ± 0.85	5.04 ± 0.27	4.88 ± 0.39	5.73 ± 0.29	0.143	0.050 (*x*/*y*)	0.206
CAS (U/mgprot)	1.54 ± 0.05	1.30 ± 0.12	1.41 ± 0.07	1.24 ± 0.14	0.406	0.068	0.736
Stomach							
LPS (U/gprot)	1.84 ± 0.23	1.15 ± 0.04	2.38 ± 0.59	2.00 ± 0.07	0.180	0.963	0.931
CAS (U/mgprot)	6.26 ± 0.95	7.32 ± 0.42	5.21 ± 0.83	6.05 ± 0.70	0.009 (*b*/*a*)	0.026 (*x*/*y*)	0.777

*Notes*: MO, *M. oleifera* supplementation; *Ba, B. amyloliquefaciens* supplementation; MO × Ba, interaction effect. Values are means and standard error of the mean (SEM). ns, no significant, *⁣*^*∗*^*P* < 0.050. Different letters indicate significant differences between the two MO adding types and *B. amyloliquefaciens* adding types, such as (*a*/*b*) in parentheses corresponds to no MO addition, MO addition, and the value of *a* is less than *b*. The other set of letters (*x*/*y*) corresponds to no *B. amyloliquefaciens* addition and *B. amyloliquefaciens* addition, and the value of *x* is less than the value of *y*.

**Table 7:** The immunity in the spleen of Nile tilapia fed test diets for 56 days.

Treatments	CON		MO		*P* values		
	CON	Ba	CON	Ba	MO	Ba	MO × Ba
*TNF-α* (pg/mL)	12.81 ± 0.52	13.78 ± 0.43	15.09 ± 0.27	14.90 ± 0.50	0.002 (*a*/*b*)	0.391	0.216
*IL-6* (pg/mL)	5.39 ± 0.11	5.70 ± 0.17	5.94 ± 0.14	6.52 ± 0.25	0.011 (*a*/*b*)	0.065	0.455
*IL-10* (pg/mL)	22.20 ± 1.88	25.25 ± 1.36	27.27 ± 1.29	24.08 ± 0.89	0.200	0.963	0.052
*C3* (pg/mL)	23.31 ± 1.33	24.58 ± 0.77	29.16 ± 0.14	28.70 ± 1.00	<0.001 (*a*/*b*)	0.668	0.364

*Notes*: MO, *M. oleifera* supplementation; Ba, *B. amyloliquefaciens* supplementation; MO × Ba, interaction effect. Values are means and standard error of the mean (SEM). ns, no significant, *⁣*^*∗*^*P* < 0.05. Different letters indicate significant differences between the two MO adding types and two Ba types, such as (*a*/*b*) in parentheses corresponds to no MO addition, MO addition, and the value of *a* is less than *b*. The other set of letters (*x*/*y*) corresponds to no Ba addition and Ba addition, and the value of *x* is less than the value of *y*.

**Table 8:** The alpha diversity of microflora in pond water and intestinal tract.

Groups	Shannon	Simpson	Ace	Chao	Coverage
CON–CON	2.33 ± 1.21A	0.26 ± 0.26C	305 ± 87.52A	261 ± 101.10A	0.9967 ± 0.00A
CON–Ba	1.79 ± 0.33A	0.36 ± 0.05C	306 ± 19.65A	243 ± 56.56A	0.9962 ± 0.00A
MO–CON	2.75 ± 1.20A	0.24 ± 0.29C	371 ± 3.95A	333 ± 53.08A	0.9925 ± 0.00A
MO–Ba	1.79 ± 0.60A	0.42 ± 0.21C	369 ± 119.53A	287 ± 76.16A	0.9964 ± 0.00A
S	3.51 ± 0.04C	0.07 ± 0.00A	609 ± 74.81B	593 ± 73.87B	0.9977 ± 0.00AB
SW	3.52 ± 0.11C	0.07 ± 0.01A	607 ± 52.14B	592 ± 51.27B	0.9979 ± 0.00B
SP	3.16 ± 0.09B	0.12 ± 0.01B	549 ± 33.19B	542 ± 39.74B	0.9975 ± 0.00AB

*Notes*: Values in the table show in the form of mean ± SEM; Values in the same row with different letters were significantly different from each other (*P* < 0.05). CON–CON stands for the no treatment diet; CON–Ba stands for 0% MO and 1.0 × 10^7^ CFU/g Ba; MO–CON stands for 10% MO and 0 CFU/g Ba; MO–Ba stands for 10% MO and 1.0 × 10^7^ CFU/g Ba. S represents the pond water at 0 day; SW is the pond water of group CON–CON and group MO–CON; SP is the pond water of group CON–Ba and group MO–Ba. The presence of different capital letters indicates a significant difference between groups.

**Table 9:** Top 20 most abundant predicted pathways of MO and Ba group intestinal microbiota in Nile tilapia.

Pathway ID	CON–CON	CON–Ba	MO–CON	MO–Ba
Metabolic pathways	4,682,208	4,174,213	4,745,730	4,361,700
Biosynthesis of secondary metabolites	2,209,745	1,810,849	2,179,152	1,915,360
Microbial metabolism in diverse environments	1,307,783	1,086,358	1,406,392	1,153,645
Biosynthesis of amino acids	935,922	726,328	837,171	752,132
Carbon metabolism	690,094	626,715	748,673	676,111
ABC transporters	562,045	510,720	557,498	498,189
Ribosome	509,757	492,115	488,196	502,658
Two-component system	494,295	477,370	456,205	459,384
Purine metabolism	411,870	411,283	417,627	423,338
Quorum sensing	343,410	312,450	349,552	311,760
Glycolysis/gluconeogenesis	315,325	296,563	318,816	308,709
Pyrimidine metabolism	297,474	328,256	282,218	328,621
Pyruvate metabolism	298,578	270,273	313,132	280,704
Amino sugar and nucleotide sugar metabolism	308,034	288,582	262,744	284,024
Porphyrin and chlorophyl metabolism	263,796	262,966	249,901	251,081
Aminoacyl–tRNA biosynthesis	248,535	255,589	249,565	258,440
Carbon fixation pathways in prokaryotes	225,694	243,065	268,152	265,767
Cysteine and methionine metabolism	247,849	249,550	235,448	246,057
Starch and sucrose metabolism	286,184	240,486	211,589	232,266
Alanine, aspartate, and glutamate metabolism	224,484	215,751	224,673	226,689

## Data Availability

All data to support the findings of this study are included in this paper.
